# American Academy of Dental Science

**Published:** 1891-06

**Authors:** 

**Affiliations:** American Academy of Dental Science


### AMERICAN ACADEMY OF DENTAL SCIENCE.

THE American Academy of Dental Science held its regular monthly meeting at the Boston Medical Library Association Rooms on February 4, 1891.

President Seabury in the chair.

The paper of the evening was by E. G. Tucker, M.D.; subject, "The Organization and Diseases of the Teeth." (For Dr. Tucker's paper, see p. 379.)

### DISCUSSION.

*Dr. Fillebrown.*—I was sitting so directly behind the speaker that I did not hear the first of the paper with sufficient clearness to be able to discuss it. I wish, however, to express my general agreement with the paper, and my pleasure in listening to the dissertation of a person of Dr. Tucker's age and experience. I wish to thank him for giving us the benefit of his thoughts to-night.

*President Seabury.*—There is one point in the paper that struck me very forcibly. We used to call decay of the teeth a vito-chemical action. Dr. Tucker has set forth very clearly that chemical action ceases when the vital action ceases; that is, that a dead tooth never decays when out of the mouth. It had not occurred to me before in that light. It is necessary to have life in order to have decay.

*Dr. Meriam.*—The rotting which the doctor speaks of is an important matter, and seems to be confined entirely to substances which go on rotting when separated from their vital connections. For instance, we find that an apple rots after its removal from the bough. The experience of the past few years has developed very clearly the medical relation of the operation of filling teeth. The change in the medical practice of to-day means an added importance to dentistry. There was a time when in nearly all diseases a low diet was prescribed. To-day, even in fevers, a patient is fed and given all the nutrition he can possibly care for. This is to prevent, as far as possible, a person coming out of a sickness in a debilitated condition. A short time ago a very learned physician said to me, referring to our specialty, "A man who fixes another so that he can eat does a great service." Medically speaking, that describes the physiological office of the dentist. "He fixes a man so that he can eat." I did not come prepared to speak, but I felt it was my place to come when Dr. Tucker was to read a paper. When he does us that honor, there is an opportunity to show the pride which some of the younger men have in him.

*Dr. Mead.*—I was saying to Dr. Tucker before the commencement of the meeting that nothing would have drawn me forty miles to-day only to hear his paper. I have known Dr. Tucker since my boyhood, as a man and as a scientific operator, and it affords me great pleasure always to meet him on these occasions, but more particularly now when, at his advanced years and with his great experience, he is equipped with those attributes which inspire every man to do good work. I am very happy to have been here this evening, and should not have come if it had not been for the pleasure of hearing him.

*President Seabury.*—The next thing in order will be "Incidents of Office Practice and Presentation of Specimens."

*President Seabury.*—I will mention a fact that was brought to my notice to-day,—that is, it is supposed to be a fact,—that aluminum foil is practical as a filling-material. It is said that it can be beaten out as thin as gold and that it is soft and pliable and will pack into a tooth as well as gold. It interested me very much and I took measures to find out more about it.

*Dr. Williams.*—I hope the president will ascertain where it can be found.

*President Seabury.*—I shall. It is a remarkable metal; it is not corroded by the oxygen of the atmosphere any more than gold is, and has other valuable qualities which make it available for our use.

*Dr. Fillebrown.*—The first aluminum manufactured for dental purposes was said to fail in the mouth on account of its not being pure. It is now being made quite pure.

*Dr. Meriam.*—I think there would be no difficulty in having any gold-beater make a foil, if a sufficient number of dentists would take it. The purer metals are, the easier they are worked. Dr. Carroll, a maker, who was on here with a furnace, produces aluminum foil, and I think I have a sample of it now which he passed around in New York. It is a soft foil to be used in cylinders. I have not attempted to use it, but think there is no cohesion, and should question whether it would be as practicable as gold.

*Dr. Williams.*—Some time ago I tried to mix aluminum with mercury, but they would not combine. Perhaps it was due to the impurity of the first preparations of aluminum, though I doubt it. I have not tried the experiment of late.

*Dr. Eddy.*—I wish to show a convenient method of having bill-heads. They are blocked and have stubs after the style of checkbooks. By having them in this shape they are always ready instead of being scattered in different drawers of your desk.

*Dr. Taft.*—I should like to ask Dr. Eddy how he itemizes a bill in case he is requested to do so by the patient?

*Dr. Eddy.*—I send them a diagram of the mouth, specifying on that the work done. The majority of them don't know a lateral from a molar, but by sending a chart they will know where the work was done. I don't suppose there are more than one or two a quarter who ask for an itemized account.

William H. Potter, D.M.D.,
*Editor American Academy of Dental Science.*

